# Lobar Cerebral Microbleeds Are Associated With Cognitive Decline in Patients With Type 2 Diabetes Mellitus

**DOI:** 10.3389/fneur.2022.843260

**Published:** 2022-03-25

**Authors:** Pengfei Shao, Hengheng Xu, Xiaoning Sheng, Ruomeng Qin, Junyi Ma, Yishan Luo, Allan Lee, Lin Shi, Lili Huang, Yue Cheng, Hui Zhao, Yun Xu

**Affiliations:** ^1^Department of Neurology, Affiliated Drum Tower Hospital, Nanjing University Medical School, Nanjing, China; ^2^Jiangsu Key Laboratory for Molecular Medicine, Nanjing University Medical School, Nanjing, China; ^3^Jiangsu Province Stroke Center for Diagnosis and Therapy, Nanjing, China; ^4^BrainNow Research Institute, Shenzhen, China; ^5^Department of Imaging and Interventional Radiology, The Chinese University of Hong Kong, Pokfulam, Hong Kong SAR, China

**Keywords:** cerebral microbleeds (CMBs), quantitative method, type 2 diabetes mellitus, cognitive impairment, MRI

## Abstract

**Purpose:**

Combined the number, volume, and location of cerebral microbleeds (CMBs), this study aimed to explore the different features of CMBs and their correlation with cognitive ability in patients with type 2 diabetes mellitus (T2DM).

**Methods:**

This study recruited 95 patients with T2DM and 80 healthy control (HC) individuals. AccuBrain^®^, an automated tool, was used to obtain the number and volume of CMBs. The scores on global cognition and five cognitive domains were derived from a battery of cognitive tests. The logistic regression and multivariate linear regression were conducted to determine the relationship between the CMBs (number, volume, and location) and cognitive ability in patients with T2DM.

**Results:**

After adjusting for several variables, the total volume of CMBs (OR = 0.332, 95%CI: 0.133–0.825, and *p* = 0.018) was independent risk factor for cognitive impairment, whereas the total number of CMBs was not (OR = 0933, 95%CI: 0.794–1.097, and *p* = 0.400). Furthermore, the volume of CMBs in lobar regions was independently associated with working memory (β = −0.239, 95%CI: −0.565 to −0.035, and *p* = 0.027). However, no significant correlation between the number of CMBs (both lobar and deep/infratentorium) and any cognitive domains was observed.

**Conclusions:**

Lobar CMBs was related with cognitive impairment in patients with T2DM and might be a potential early warning signal. Compared with the counting analysis, the quantitative method offered a more sensitive and objective measurement for studying imaging features of CMBs.

## Introduction

People with diabetes are 1.5–2.0 times more likely than those without diabetes to have cognitive impairment (1). In patients (aged 65–74 years) with T2DM, the prevalence of cognitive impairment is 13% and in patients aged 75 years or older is 24% (2). Diabetes guidelines also recommend screening for cognitive impairment in people with diabetes over 65 years of age and early intervention (3, 4). This high prevalence and the associated morbidity highlight the importance of studying the pathophysiological mechanisms underlying cognitive impairment in T2DM. Till now, the etiology is incompletely known, but vascular disease is likely to play an important role (5). As a known risk factor, T2DM affected both large and small vessels. Microvascular complications of T2DM appear in the retina, peripheral nervous system, the kidney, and also the brain (6). Some studies suggested that cerebral small vessel disease (CSVD) may serve as a key factor in the development of T2DM-related cognitive impairment.

Cerebral microbleeds, a neuroimaging maker of CSVD, are defined as small, low signal, circular lesions on sensitivity weighted imaging (SWI) and T2^*^weighted gradient echo (T2^*^GRE) sequences, cannot be observed on computed tomography (CT). CMBs result from focal leakages of small vessels (generally they are 2–5 mm in diameter, but up to 10 mm) (6). Of note, the distribution of CMBs is related to the likely underlying pathophysiological mechanisms. Generally, CMBs in the deep/infratentorium (basal ganglia, thalamus, corpus callosum, brain stem, and cerebellum) are typically associated with hypertension, whereas the CMBs in the lobar (frontal, parietal, temporal, and occipital) may suggest cerebral amyloid angiopathy (CAA) (7).

**Figure 1:**
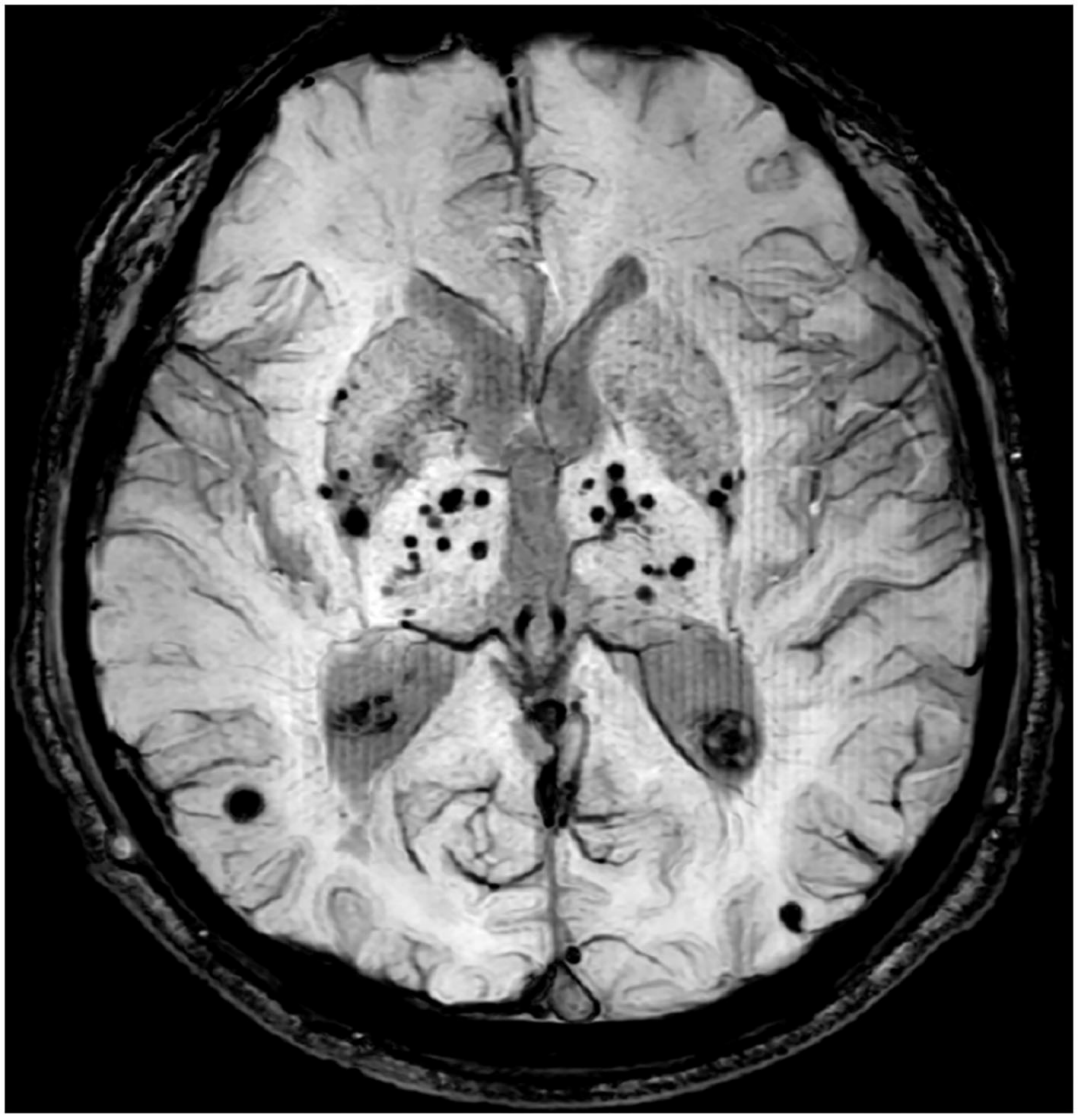
CMBs lesions with different size in SWI image.

Several studies used manual counting method to assess the burden of CMBs in patients with T2DM, the results showed neither the presence nor the number of CMBs were associated with cognitive ability (8–10). Visual rating scales offer a relatively easy and quick method to measure CMBs, but its limitation is also obvious (e.g., time-consuming and low accuracy). Alternatively, some automatic detection methods have been developed to better identify and count CMBs. However, as the size of different lesions may vary several times (Figure 1), so either the manual counting method or automatic detection method was easily hampered by the floor and ceiling effects (11, 12). In contrast, the advantages of quantitative image analysis have been widely reported in a large number of studies. Until now, multiple methods are available to quantify CMBs, and these methods vary from fully manual to fully automatic. For example, a study multiplied area of each lesion by the slice thickness to get the volume of CMBs (13). Some traditional tools, like FSL (https://www.fmrib.ox.ac.uk/fsl), and FreeSurfer (https://surfer.nmr.mgh.harvard.edu/) were widely used to obtain the volume of CMBs (14, 15). Morrison et al. (16) developed an automatic tool that could detect CMBs for volume quantification. But those methods still are time-consuming or low accuracy. Currently, some automatic quantification tools were used to calculate the burden of CSVD image marker, whose robustness and efficiency have been greatly improved with the aid of deep learning techniques. For example, AccuBrain^®^ could support automatic quantification of brain atrophy, white matter hyperintensity (WMH), and CMBs (17–19).

For the first time, we utilized neuropsychological tests and the magnetic resonance imaging (MRI) technology to investigate the cognitive function and the number, volume, and location features of CMBs in T2DM subjects. The main aim of our study was to test the hypothesis that impaired cognitive function in T2DM subjects may be partially due to CMBs. We further tested whether this hypothesized effect could be explained with the different features of CMBs.

## Methods and Materials

### Participants

A total of 175 right-handed subjects were recruited from January 2017 to June 2019 at the Neurology Department of the Affiliated Drum Tower Hospital of Nanjing University Medical School. This study was carried out in accordance with the latest version of the Declaration of Helsinki and approved by the Drum Tower Hospital Research Ethics Committee. Written informed consent was provided from each subject. Participants were divided into two groups: the T2DM group (*n* = 95) and the HC group (*n* = 80). T2DM diagnosis was based on the latest American Diabetes Association criteria (20). Referencing previous literature, subjects with Mini-Mental State Examination (MMSE)/Montreal Cognitive Assessment (MoCA) scores lower than education-adjusted norms were defined as the cognitive impairment (Table 1) (21). The HC group had no cognitive impairments and no history of T2DM. The exclusion criteria for all subjects were as follows: (1) age < 45 years, (2) diabetes other than T2DM (e.g., prediabetes and T1DM), (3) the acute metabolic complications such as diabetic ketoacidosis, (4) the major macrovascular and microvascular complications such as stroke, cardiovascular disease, renal insufficiency, and diabetic neuropathy, (5) history of other neurological diseases that may affect cognition (e.g., Alzheimer's disease, epilepsy, and so on), and (6) contraindications for MRI and/or inability to undergo cognitive tests or failed to complete the assessment.

**Table 1:** The cutoffs of cognitive impairment for all participants.

**Cognitive test**	**Education (years)**	**Borderlines of cognitive impairment (scores)**
MMSE	0	≤ 19
	1–6	≤ 22
	>6	≤ 26
MoCA	0	≤ 13
	1–6	≤ 19
	7–12	≤ 24
	>12	≤ 25

*MMSE, Mini-Mental State Examination; MoCA, Montreal Cognitive Assessment*.

### Neuropsychological Evaluation

Neuropsychological tests were evaluated by an experienced psychiatrist on the same day that patients underwent MRI scans. As used in previous studies, the Chinese version of MMSE and the Beijing version of MoCA were used to evaluate global cognition (21, 22). Additionally, the five cognitive domains and each raw test were (1) working memory: the auditory verbal learning test-long time delay recall (AVLT-LTDR) test and the visual reproduction-delay recall (VR-DR) test, (2) visual-spatial ability: the VR-copy (VRC) test and the clock drawing test (CDT), (3) executive function: the trail making test (TMT)B-A and the Stroop color and word test (SCWT)C-B, (4) language ability: the category verbal fluency test (CVFT) and the Boston naming test (BNT), and (5) processing speed: the TMTA and the SCWTB. Each raw test score was standardized into *z*-scores and averaged to obtain one composite *z-*score, which represents the overall performance per cognitive domain. It is noteworthy that the results from the SCWT and TMT, which represent time, were inversely transformed to maintain consistency.

### MRI Data Acquisition

All MRI scans were acquired using 3.0-T scanner (Philips, the Netherlands) equipped with a 32-channel head coil. The examination protocol included a 3-dimensional T1-weighted sequence (3D-T1) [repetition time (TR) = 9.8 ms, echo time (TE) = 4.6 ms, flip angle (FA) = 8°, number of slices = 192, acquisition matrix = 256 × 256, thickness = 1.0 mm, and FOV = 250 × 250 mm^2^], a 3-dimensional fluid-attenuated inversion recovery (FLAIR) sequence [TR = 4,500 ms, TE = 333 ms, number of slices = 200, voxel size = 0.95 × 0.95 × 0.95 mm^3^, and acquisition matrix = 270 × 260], and a SWI sequence (TR = 31 ms, TE = 7.2 ms, FA = 17°, number of slices = 162, acquisition matrix = 480 × 480, and FOV = 230 × 230 mm^2^).

### Image Analysis

#### Cerebral Microbleeds

AccuBrain^®^ includes a program to detect CMBs. It has been validated in independent dataset (sensitivity is 0.89 and precision is 0.47), which is at the same level of the state-of-the-art automated CMB detection methods. CMBs were detected through a fully connected network (FCN) trained using deep learning technique on SWI images, the FCN was trained using 274 SWI images and their manual CMBs labels. Given a testing SWI MRI, the network outputted a probability map showing CMB positions. Based on the probability map, a CMB segmentation image could be produced based on the optimal threshold chosen in the training process. On the basis of segmentation results, multiplied the size of voxels by the number, and then we got the volume of the CMBs. Due to the variation of the intensities and shape of CMBs on SWI images, designing effective and robust features can be quite challenging. This can be overcome by using convolutional neural network-based deep learning techniques, and it is more effective on false-positive reduction compare to convention algorithm such as Frangi filter and radial transform. Due to the large dataset and expensive manual annotation efforts, we took automated CMB detection method to keep objectivity.

#### White Matter Hyperintensity

The LST toolbox (https://www.statistical-modelling.de/lst.html) based on SPM12 (http://www.fil.ion.ucl.ac.uk/spm/software/spm12/) software was used to calculate the volume of WMH. First, the 3D-T1 images were segmented into the three main tissue classes (gray matter, white matter, and cerebrospinal fluid). Second, above information is combined with the coregistered T2 FLAIR intensities to calculate lesion belief maps. Third, an initial binary lesion map is obtained by thresholding these maps with a pre-chosen threshold (κ = 0.30) and then grown along voxels that appear hyperintense on the T2 FLAIR image. Finally, a lesion probability map is obtained. It worth noting that the κ-value was determined by the visual inspection of the results by three experienced raters.

### Clinical Variables

Age, sex, education, height and weight, and body mass index (BMI) [(weight in kg)/(height in m)^2^], and T2DM duration were obtained from questionnaires and medical records. Hypertension was defined as a systolic arterial blood pressure (SBP) ≥140 mmHg, diastolic arterial blood pressure (DBP) ≥90 mmHg, or a history of arterial hypertension or indicated by a record of anti-hypertensive therapy. Smokers were defined as those who smoked at least once (one or more cigarettes at a time) in the preceding year, and drinkers were defined as those who drank alcohol on more than 2 days (one or more glasses at a time) a week. Levels of glycosylated hemoglobin (HbA1c), fasting plasma glucose (FPG), total cholesterol (TC), triglyceride (TG), low-density lipoprotein (LDL), and high-density lipoprotein (HDL) were measured in a standard laboratory.

### Statistical Analysis

Data are represented as mean ± SD or *n* (%). A log_10_-transformation was applied to the volume of CMBs and WMH to improve the normal distribution. Independent samples *t*-test, Mann–Whitney *U*-test, and χ^2^ test was conducted to estimate group differences. Covariates that were significant in univariate analysis were entered into logistic regression analysis and multiple linear regression in T2DM group (*n* = 95). Both age and education are known to be associated with cognitive function, and they were also included in the regression analyses as covariates. The level of significance was set at *p* < 0.05. In addition, we applied correction for multiple comparison (Bonferroni correction, *p* < 0.05/5) in multiple linear regression. When the Bonferroni correction was too stringent, and not to miss some exploratory interesting results, the statistical threshold for significance was set as *p* < 0.05 (without Bonferroni correction). When we observed a significant association between CMBs (number or volume) and cognitive function in multiple linear regression in the T2DM group, we would further explore the same associations in the HC group (*n* = 80). All statistical analyses were carried out using SPSS version 22 (IBM Corp., Armonk, NY, USA).

## Result

### Clinical Data

There were 95 participants in the T2DM group and 80 in the HC group. Group characteristics and comparisons are listed in Table 2. T2DM participants reported a disease duration of 9.06 ± 13.00 years (mean ± SD). T2DM participants had higher BMI and HbA1c values, larger volume of CMBs (total, lobar, and deep/infratentorium) and WMH, and lower levels of TC, HDL, and LDL. In addition, T2DM participants performed worse than the HC group participants in several cognitive tests or domains (MMSE, MoCA, and working memory). No significant differences were observed in age, gender, education, TG, hypertension, smoking, drinking, the number of CMBs, executive function, processing speed, language function, and visual-spatial ability between the two groups.

**Table 2:** Clinical data for all subjects.

	**T2DM (*n* = 95)**	**HC (*n* = 80)**	**T/χ^2^/Z**	***p*-value**
Age (years)	63.85 ± 9.02	62.63 ± 9.42	0.870	0.386
Gender (male, %)	45 (47.37)	39 (48.75)	0.03	0.855
Education (years)	10.43 ± 4.26	11.71 ± 4.50	−1.932	0.055
BMI (kg/m^2^)	23.50 ± 2.53	22.16 ± 2.20	3.730	<0.001^*^
T2DM duration (years)	9.06 ± 13.00	–	–	–
HbA1c (%)	6.77 ± 1.34	5.19 ± 0.55	9.814	<0.001^*^
TC (mmol/L)	4.09 ± 1.06	4.58 ± 1.29	−2.609	0.010^*^
TG (mmol/L)	1.40 ± 0.84	1.44 ± 0.77	−0.316	0.752
HDL (mmol/L)	1.22 ± 0.40	1.37 ± 0.47	−2.131	0.035^*^
LDL (mmol/L)	2.32 ± 0.82	2.68 ± 1.02	−2.438	0.016^*^
Hypertension (*n*, %)	63 (66.31)	47 (58.75)	1.065	0.302
Smoking (*n*, %)	21 (22.34)	15 (18.75)	0.340	0.560
Drinking (*n*, %)	18 (19.57)	16 (20.00)	0.005	0.943
Volume of WMH (cm^3^)	7.81 ± 13.20	4.53 ± 8.37	−2.727	0.006^*^
Volume of WMH^**#**^	0.53 ± 0.57	0.26 ± 0.61	2.950	0.004^*^
Total number of CMBs (*n*)	3.52 ± 12.71	0.71 ± 1.16	1.972	0.051
lobar regions (*n*)	2.46 ± 8.53	0.59 ± 1.04	1.952	0.053
Deep/infratentorium (*n*)	1.06 ± 4.36	0.13 ± 0.37	1.919	0.057
Total volume of CMBs (mm^3^)	40.37 ± 247.7	3.24 ± 7.73	−5.199	<0.001^*^
lobar regions (mm^3^)	26.48 ± 166.1	2.12 ± 2.46	−4.587	<0.001^*^
Deep/infratentorium (mm^3^)	14.66 ± 82.84	2.06 ± 7.42	−3.651	<0.001^*^
Total volume of CMBs^**#**^	0.72 ± 0.70	0.23 ± 0.40	5.373	<0.001^*^
lobar regions^#^	0.55 ± 0.64	0.17 ± 0.32	4.847	<0.001^*^
Deep/infratentorium^#^	0.30 ± 0.58	0.06 ± 0.26	3.400	<0.001^*^
MMSE	27.76 ± 2.40	28.70 ± 1.33	−3.126	0.002^*^
MoCA	23.37 ± 3.97	26.09 ± 2.09	−5.509	<0.001^*^
Z-Executive function	0.84 ± 11.30	0.77 ± 9.82	0.043	0.966
Z-Processing speed	−1.83 ± 20.98	1.87 ± 29.07	−0.975	0.331
Z-Working memory	−0.18 ± 0.80	0.213 ± 0.64	−3.537	0.001^*^
Z-Language function	−0.08 ± 0.75	0.11 ± 0.59	−1.866	0.064
Z-Visual-spatial ability	−0.08 ± 0.8	0.13 ± 0.62	−1.832	0.069

*T2DM, type 2 diabetes mellitus; HC, type 2 diabetes mellitus; BMI, body mass index; HbA1c, Levels of glycosylated hemoglobin; TC, total cholesterol; TG, triglyceride; HDL, high-density lipoprotein; LDL, low-density lipoprotein; WMH, white matter hyperintensities; CMBs, cerebral microbleeds*.

# *indicates log_10_-transformation*,

* *indicates a statistical difference between T2DM group and HC group, p < 0.05*.

### The Logistic Regression in T2DM Group

Because of the linear correlation between the number and volume of CMBs, multivariate logistic regression was conducted separately to explore the independent risk factors of cognitive impairment in the T2DM group (*n* = 95). After adjusting for age, education, BMI, HbA1c, TC, HDL, LDL, and volume of WMH, the total volume of CMBs (OR = 0.332, 95% CI: 0.133–0.825, and *p* = 0.018) was independent risk factors for cognitive impairment, whereas the total number of CMBs was not (OR = 0.933, 95% CI: 0.794–1.097, and *p* = 0.400) (Table 3).

**Table 3:** Correlations between CMBs and cognitive impairment in the T2DM group.

	**OR**	**95% CI**	***p***
Total number of CMBs	0.933	0.794 to 1.097	0.400
Total volume of CMBs^#^	0.332	0.133 to 0.825	0.018^*^

*OR, odd ratio; CI, confidence interval; CMBs, cerebral microbleeds*.

# *indicates log_10_-transformation*,

* *indicates p < 0.05*.

*Values are adjusted for age, education, BMI, HbA1c, TC, HDL, LDL, and volume of WMH*.

### The Multivariate Linear Regression in the T2DM and HC Groups

Multivariate linear regression was conducted separately to explore the correlation between CMBs in different regions and specific cognitive domains in the T2DM group (*n* = 95). After adjusting for age, education, BMI, HbA1c, TC, HDL, LDL, and volume of WMH, no significant correlation between the number of CMBs (lobar regions or deep/infratentorium) and any cognitive domains was observed (Table 4). However, the volume of CMBs in lobar regions was independently associated with working memory (β = −0.239, 95%CI: −0.565 to −0.035, *p* = 0.027) (Table 5). In addition, there was no significant correlation between volume of CMBs (lobar regions or deep/infratentorium) and any cognitive domains in the HC groups (Table 6).

**Table 4:** Multiple linear regression analysis in the T2DM group (number of CMBs).

**Cognitive variable**	**Lobar regions**		**Deep/infratentorium**	
	**β (95% CI)**	***P*-value**	**β (95% CI)**	***P-*value**
Z-Executive function	−0.052 (−0.367 to 0.229)	0.647	−0.045 (−0.704 to 0.472)	0.696
Z-Processing speed	0.020 (−0.487 to 0.586)	0.855	0.033 (−0.903 to 1.216)	0.769
Z-Working memory	−0.154 (−0.034 to 0.005)	0.143	−0.132 (−0.063 to 0.014)	0.216
Z-Language function	0.091 (−0.008 to 0.024)	0.327	0.062 (−0.021 to 0.042)	0.504
Z-Visual-spatial ability	0.071 (0.015 to 0.029)	0.512	0.038 (−0.036 to 0.051)	0.726

*CI, Confidence interval; the statistical threshold for significance was set as p < 0.05. Values are adjusted for age, education, BMI, HbA1c, TC, HDL, LDL, and volume of WMH*.

**Table 5:** Multiple linear regression analysis in T2DM group (volume of CMBs).

**Cognitive variable**	**Lobar regions**		**Deep/infratentorium**	
	**β (95% CI)**	***p*-value**	**β (95% CI)**	***p*-value**
Z-Executive function	−0.223 (−7.983 to 0.087)	0.055	−0.106 (−6.984 to 2.844)	0.405
Z-Processing speed	0.036 (−6.221 to 8.631)	0.749	0.051 (−7.029 to 10.715)	0.681
Z-Working memory	−0.239 (−0.565 to −0.035)	0.027^*^	−0.064 (−0.414 to 0.237)	0.590
Z-Language function	0.103 (−0.100 to 0.343)	0.279	0.047 (−0.205 to 0.327)	0.651
Z-Visual-spatial ability	−0.051 (−0.372 to 0.232)	0.645	−0.007 (−0.373 to 0.351)	0.952

*CI, Confidence interval*;

* *indicates p < 0.05. Values are adjusted for age, education, BMI, HbA1c, TC, HDL, LDL, and volume of WMH*.

**Table 6:** Multiple linear regression analysis in the HC group (volume of CMBs).

**Cognitive variable**	**Lobar regions**		**Deep/infratentorium**	
	**β (95% CI)**	***p*-value**	**β (95% CI)**	***p*-value**
Z-Executive function	0.188 (−2.191 to 14.676)	0.144	−0.092 (−13.618 to 6.732)	0.501
Z-Processing speed	−0.011 (−24.60 to 22.40)	0.926	−0.838 (−50.45 to 1.338)	0.063
Z-Working memory	−0.081 (−0.605 to 0.298)	0.499	0.106 (−0.308 to 0.763)	0.399
Z-Language function	0.101 (−0.298 to 0.678)	0.439	−0.011 (−0.608 to 0.560)	0.935
Z-Visual-spatial ability	0.031 (−0.441 to 0.566)	0.806	−0.041 (−0.689 to 0.508)	0.764

*CI, Confidence interval; the statistical threshold for significance was set as p < 0.05*.

*Values are adjusted for age, education, BMI, HbA1c, TC, HDL, LDL, and volume of WMH*.

## Discussion

To our knowledge, this was the first study to do quantitative analysis about the imaging features of CMBs in patients with T2DM. Our study found that compared with the number of CMBs, the volume of CMBs was the independent risk factors for cognitive impairment in patients with T2DM. Furthermore, the volume of CMBs in lobar regions was negatively correlated with working memory. Remarkably, there was no significant correlation between the number of different location and cognitive ability in patients with T2DM.

Our study suggested that the total number of CMBs does not correlate with cognitive impairment in patients with T2DM. Similarly, a cross-sectional study included 350 T2DM participants and 363 participants without T2DM suggested that CMBs do not seem to be associated with cognition (9). Another research that employed high fields (7-T) in patients with T2DM further confirmed that neither the presence nor the number of CMBs were associated with cognitive ability (8). A research included 4,206 participants of the Reykjavik study reported that CMBs did not mediate the association of diabetes with cognitive ability (10). However, the quantitative analysis showed an opposite conclusion. Our study suggested that the total volume of CMBs was the independent risk factors for cognitive impairment in patients with T2DM. Similar to our results, the Leukoaraiosis And DISability (LADIS) study included 618 elderly reported the volume of WMH was more sensitive than visual rating scores with respect to memory symptoms, and the number of lesions was not sensitive to explore associations with clinical variables (11). Those results suggest that associations between CMBs and cognition may depend on the method applied for measuring the burden of CMBs. Visual rating scales relied heavily on the human eye to detect and count CMB lesions, which carried several problems. First, it was time-consuming and decreased the intra- and inter-rater reliability. Second, scores from different visual rating scales were not comparable. Third, in progressing disease, the merge of different lesions caused a decrease in the total number of lesions (11). In addition, either manual visual rating scales or automatic detection method displays the ceiling effects, which made it difficult to draw firm conclusion between CMBs and clinical variables. Obviously, counting analysis (visual rating scales or automatic detection) remains inferior to quantitative methods. For example, many studies on WMH-confirmed volumetric measurements were more reliable and objective compared with the visual rating scale, especially in longitudinal studies (23, 24). Detailed evaluation for volume of CMBs by these new methods may provide vital information on the etiology, progression, and prognosis of diseases.

Our study suggested that the volume of CMBs was the independent risk factors for cognitive impairment. However, little is known about how CMBs effected the cognitive ability of patients with T2DM. There might have several explanations: (1) CMB lesions could directly damage the surrounding brain tissue, disrupt the functional connections of cortical and subcortical tracts, lead to the damage of neural networks, which are important to maintain normal cognition, and whether the hemosiderin deposition is sufficient to result in tract degeneration still need further study (7, 25); (2) CMBs could induce functional disturbances in nearby neurons (26); and (3) CMBs ruined the blood–brain barrier, and greater volume of CMBs meant more widely microvascular damage, gliosis, hypoperfusion, and so on (27). Our study further observed that the volume of CMBs in lobar regions was negatively correlated with working memory, whereas the volume of CMBs in deep/infratentorium does not correlated with any cognitive domains. Similarly, the I-Lan Longitudinal Aging Study (ILAS) suggested that strictly lobar CMBs correlated with global cognition and visuospatial executive functions, whereas deep/infratentorial CMBs were not associated with any cognitive domains (28). Another study from Rotterdam indicated that lobar CMBs were associated with a dysfunction in memory, executive function, and processing speed (29). The exact pathophysiological mechanism, which underlie the relationship between lobar CMBs and cognitive domains, is not clear. Lobar CMBs have been considered to be the brain disorders caused by CAA. In this scenario, vascular amyloid-β (Aβ) damaged the neurovascular unit, then resulted in hypoperfusion, impaired vascular autoregulation or reactivity, and even microinfarction (30, 31). Lobar CMBs might be the “tip of iceberg” of widespread CAA-related dysfunction in cortical small vessels. Of note, lobar CMBs might serve as an intriguing link between cerebrovascular and neurodegenerative pathology (32). In addition, the correlation of lobar CMBs with working memory is partial because that lobar CMBs had a predilection for the temporal lobes (29). In contrast, deep/infratentorial CMBs were mainly distributed in the basal ganglia and internal capsule, affecting motor function. In addition, we did not observe a significant correlation between volume of CMBs (lobar regions or deep/infratentorium) and any cognitive domains in the HC group. Participants in the HC group usually have normal performance in global cognition or several cognitive domains, so it is difficult to observe a significant correlation between CMBs and cognition in the HC group.

Major strengths of our study include the advanced tool and MRI sequence for the detection and quantitative analysis of CMB lesions. Most studies detected CMBs by GRE sequence; however, the sensitivity of SWI sequence for CMBs was almost four times higher (33). AccuBrain^®^ is a cloud-based computing tool, which only requires MRI scans as input with no other tunable parameters. Compared with other quantification tools/methods, AccuBrain^®^ showed higher accuracy and efficiency, it could efficiently quantify the volumes of various brain structure in about 20 min. Notably, there are some limitations to this study. First, the nature of this study is a single-center data and a small sample set, so we cannot obtain firm causal relationship, multicenter and expanding datasets are needed in the future. Second, the cross-sectional design of the present research limits the ability to catch the more real dynamic process of CMB changes, and longitudinal studies on cognitive changes of patients with T2DM are necessary. Third, the present sample of patients with T2DM and normal participants was recruited based on clinical criteria, we lacked pathological biomarkers to define the etiologic subtypes of CMBs strictly that might be helpful to offer a comprehensive insight into the pathologic role of CMBs in T2DM. Fourth, the association between volume of lobar CMBs and working memory in the T2DM group was not significant after correction for multiple comparison (Bonferroni correction, *p* < 0.05/5). However, in order to not miss some exploratory interesting results, we used more liberal uncorrected statistical thresholds (*p* < 0.05, without Bonferroni correction). More participants are needed in the future to draw more accurate and convincing conclusions.

## Conclusion

In summary, our study indicated that the CMBs are associated with cognitive decline in patients with T2DM. Specifically, the volume of CMBs in lobar regions is independently related with working memory, which might be a potential signal for early identification and intervention. Quantitative analysis should be a more useful method to explore the pathological role of CMBs in T2DM-related cognitive impairment.

## Data Availability Statement

The original contributions presented in the study are included in the article/supplementary material, further inquiries can be directed to the corresponding author/s.

## Ethics Statement

The studies involving human participants were reviewed and approved by Drum Tower Hospital Research Ethics Committee, Affiliated Drum Tower Hospital, Nanjing University Medical School, Nanjing, Jiangsu. The patients/participants provided their written informed consent to participate in this study.

## Author Contributions

HZ and YX: conceptualization and methodology. PS and HX: MRI data acquisition and thesis writing. XS, RQ, and JM: cognitive scale examination. YC: statistics. YL, AL, and LS: software and validation. All authors contributed to the article and approved the submitted version.

## Funding

This study was funded by grants from the National Key Research and Development Program of China (2016YFC1300500-504), the National Natural Science Foundation of China (81630028), the Key Research and Development Program of Jiangsu Province of China (BE2016610), the Jiangsu Province Key Medical Discipline (ZDXKA2016020), the National Natural Science Foundation of China (81771157), the Jiangsu Province 333 project, and the Nanjing Medical Science and Technique Development Foundation (ZKX13020).

## Conflict of Interest

The authors declare that the research was conducted in the absence of any commercial or financial relationships that could be construed as a potential conflict of interest.

## Publisher's Note

All claims expressed in this article are solely those of the authors and do not necessarily represent those of their affiliated organizations, or those of the publisher, the editors and the reviewers. Any product that may be evaluated in this article, or claim that may be made by its manufacturer, is not guaranteed or endorsed by the publisher.

## Abbreviations

BMI: body mass index
CAA: cerebral amyloid angiopathy
CMBs: cerebral microbleeds
CSVD: cerebral small vessel disease
CT: computed tomography
FLAIR: fluid attenuated inversion recovery
FPG: fasting plasma glucose
HbA1c: glycosylated hemoglobin
HC: healthy control
HDL: high-density lipoprotein
LDL: low-density lipoprotein
MMSE: Mini-Mental State Examination
MoCA: Montreal Cognitive Assessment
MRI: magnetic resonance imaging
SWI: sensitivity weighted imaging
T2^*^GRE: T2^*^weighted gradient echo
T2DM: type 2 diabetes mellitus
WMH: white matter hyperintensity.

## References

[1] Cukierman-Yaffe T Gerstein HC Colhoun HM Diaz R García-Pérez L-E Lakshmanan M. Effect of dulaglutide on cognitive impairment in type 2 diabetes: an exploratory analysis of the REWIND trial. Lancet Neurol. (2020) 19:582–90. 10.1016/S1474-4422(20)30173-3 32562683

[2] Feil DG Rajan M Soroka O Tseng CL Miller DR Pogach LM. Risk of hypoglycemia in older veterans with dementia and cognitive impairment: implications for practice and policy. J Am Geriatr Soc. (2011) 59:2263–72. 10.1111/j.1532-5415.2011.03726.x 22150156

[3] American Diabetes Association. 12. Older adults: standards of medical care in diabetes-2020. Diabetes Care. (2020) 43(Suppl. 1):S152–S62. 10.2337/dc20-S012 31862755

[4] Rosenzweig JL Conlin PR Gonzalvo JD Kutler SB Maruthur NM Solis P. 2019 Endocrine society measures set for older adults with type 2 diabetes who are at risk for hypoglycemia. J Clin Endocrinol Metab. (2020) 105:969–90. 10.1210/clinem/dgz250 31825487 PMC7753052

[5] Biessels GJ Nobili F Teunissen CE Simó R Scheltens P. Understanding multifactorial brain changes in type 2 diabetes: a biomarker perspective. Lancet Neurol. (2020) 19:699–710. 10.1016/S1474-4422(20)30139-3 32445622

[6] Wardlaw JM Smith EE Biessels GJ Cordonnier C Fazekas F Frayne R. Neuroimaging standards for research into small vessel disease and its contribution to ageing and neurodegeneration. Lancet Neurol. (2013) 12:822–38. 10.1016/S1474-4422(13)70124-8 23867200 PMC3714437

[7] Yakushiji Y Werring DJ. Cerebrovascular disease: lobar cerebral microbleeds signal early cognitive impairment. Nat Rev Neurol. (2016) 12:680–2. 10.1038/nrneurol.2016.179 27857119

[8] Brundel M Reijmer YD van Veluw SJ Kuijf HJ Luijten PR Kappelle LJ. Cerebral microvascular lesions on high-resolution 7-Tesla MRI in patients with type 2 diabetes. Diabetes. (2014) 63:3523–9. 10.2337/db14-0122 24760137

[9] Moran C Phan TG Chen J Blizzard L Beare R Venn A. Brain atrophy in type 2 diabetes: regional distribution and influence on cognition. Diabetes Care. (2013) 36:4036–42. 10.2337/dc13-0143 23939539 PMC3836136

[10] Qiu C Sigurdsson S Zhang Q Jonsdottir MK Kjartansson O Eiriksdottir G. Diabetes, markers of brain pathology and cognitive function: the Age, Gene/Environment Susceptibility-Reykjavik Study. Ann Neurol. (2014) 75:138–46. 10.1002/ana.24063 24243491 PMC4540233

[11] van Straaten EC Fazekas F Rostrup E Scheltens P Schmidt R Pantoni L. Impact of white matter hyperintensities scoring method on correlations with clinical data: the LADIS study. Stroke. (2006) 37:836–40. 10.1161/01.STR.0000202585.26325.74 16439704

[12] Wardlaw JM Ferguson KJ Graham C. White matter hyperintensities and rating scales-observer reliability varies with lesion load. J Neurol. (2004) 251:584–90. 10.1007/s00415-004-0371-x 15164192

[13] Wang X Wei XE Li MH Li WB Zhou YJ Zhang B. Microbleeds on susceptibility-weighted MRI in depressive and non-depressive patients after mild traumatic brain injury. Neurol Sci. (2014) 35:1533–9. 10.1007/s10072-014-1788-3 24740482

[14] Chung CP Chen JW Chang FC Li WC Lee YC Chen LF. Cerebral microbleed burdens in specific brain regions are associated with disease severity of cerebral autosomal dominant arteriopathy with subcortical infarcts and leukoencephalopathy. J Am Heart Assoc. (2020) 9:e016233. 10.1161/JAHA.120.016233 32552418 PMC7670534

[15] Lawrence TP Pretorius PM Ezra M Cadoux-Hudson T Voets NL. Early detection of cerebral microbleeds following traumatic brain injury using MRI in the hyper-acute phase. Neurosci Lett. (2017) 655:143–50. 10.1016/j.neulet.2017.06.046 28663054 PMC5541760

[16] Morrison MA Payabvash S Chen Y Avadiappan S Shah M Zou X. A user-guided tool for semi-automated cerebral microbleed detection and volume segmentation: evaluating vascular injury and data labelling for machine learning. Neuroimage Clin. (2018) 20:498–505. 10.1016/j.nicl.2018.08.002 30140608 PMC6104340

[17] Guo C Niu K Luo Y Shi L Wang Z Zhao M. Intra-scanner and inter-scanner reproducibility of automatic white matter hyperintensities quantification. Front Neurosci. (2019) 13:679. 10.3389/fnins.2019.00679 31354406 PMC6635556

[18] Liu S Hou B Zhang Y Lin T Fan X You H. Inter-scanner reproducibility of brain volumetry: influence of automated brain segmentation software. BMC Neurosci. (2020) 21:35. 10.1186/s12868-020-00585-1 32887546 PMC7472704

[19] Mai Y Yu Q Zhu F Luo Y Liao W Zhao L. AD resemblance atrophy index as a diagnostic biomarker for Alzheimer's disease: a retrospective clinical and biological validation. J Alzheimers Dis. (2021) 79:1023–32. 10.3233/JAD-201033 33459705

[20] Harreiter J Roden M. [Diabetes mellitus-Definition, classification, diagnosis, screening and prevention (Update 2019)]. Wien Klin Wochenschr. (2019) 131(Suppl. 1):6–15. 10.1007/s00508-019-1450-4 30980151

[21] Huang L Chen X Sun W Chen H Ye Q Yang D. Early segmental white matter fascicle microstructural damage predicts the corresponding cognitive domain impairment in cerebral small vessel disease patients by automated fiber quantification. Front Aging Neurosci. (2020) 12:598242. 10.3389/fnagi.2020.598242 33505302 PMC7829360

[22] Gu Y Liu R Qin R Chen X Zou J Jiang Y. Characteristic changes in the default mode network in hypertensive patients with cognitive impairment. Hypertens Res. (2019) 42:530–40. 10.1038/s41440-018-0176-4 30573810

[23] Admiraal-Behloul F van den Heuvel DM Olofsen H van Osch MJ van der Grond J van Buchem MA. Fully automatic segmentation of white matter hyperintensities in MR images of the elderly. Neuroimage. (2005) 28:607–17. 10.1016/j.neuroimage.2005.06.061 16129626

[24] Melazzini L Vitali P Olivieri E Bolchini M Zanardo M Savoldi F. White matter hyperintensities quantification in healthy adults: a systematic review and meta-analysis. J Magn Reson Imaging. (2021) 53:1732–43. 10.1002/jmri.27479 33345393

[25] Werring DJ Frazer DW Coward LJ Losseff NA Watt H Cipolotti L. Cognitive dysfunction in patients with cerebral microbleeds on T2^*^-weighted gradient-echo MRI. Brain. (2004) 127(Pt 10):2265–75. 10.1093/brain/awh253 15282216

[26] Charidimou A Werring DJ. Cerebral microbleeds and cognition in cerebrovascular disease: an update. J Neurol Sci. (2012) 322:50–5. 10.1016/j.jns.2012.05.052 22717258

[27] Moran C Beare R Phan T Starkstein S Bruce D Romina M. Neuroimaging and its relevance to understanding pathways linking diabetes and cognitive dysfunction. J Alzheimers Dis. (2017) 59:405–19. 10.3233/JAD-161166 28527209

[28] Chung CP Chou KH Chen WT Liu LK Lee WJ Chen LK. Strictly lobar cerebral microbleeds are associated with cognitive impairment. Stroke. (2016) 47:2497–502. 10.1161/STROKEAHA.116.014166 27625380

[29] Akoudad S Wolters FJ Viswanathan A de Bruijn RF van der Lugt A Hofman A. Association of cerebral microbleeds with cognitive decline and dementia. JAMA Neurol. (2016) 73:934–43. 10.1001/jamaneurol.2016.1017 27271785 PMC5966721

[30] Gorelick PB Scuteri A Black SE Decarli C Greenberg SM Iadecola C. Vascular contributions to cognitive impairment and dementia: a statement for healthcare professionals from the American Heart Association/American Stroke Association. Stroke. (2011) 42:2672–713. 10.1161/STR.0b013e3182299496 21778438 PMC3778669

[31] Iadecola C. The overlap between neurodegenerative and vascular factors in the pathogenesis of dementia. Acta Neuropathol. (2010) 120:287–96. 10.1007/s00401-010-0718-6 20623294 PMC3001188

[32] Fotuhi M Hachinski V Whitehouse PJ. Changing perspectives regarding late-life dementia. Nat Rev Neurol. (2009) 5:649–58. 10.1038/nrneurol.2009.175 19918254

[33] Pantoni L. Cerebral small vessel disease: from pathogenesis and clinical characteristics to therapeutic challenges. Lancet Neurol. (2010) 9:689–701. 10.1016/S1474-4422(10)70104-6 20610345

